# Advances in Waste Materials’ Valorization

**DOI:** 10.3390/ma19122459

**Published:** 2026-06-08

**Authors:** Natalia Howaniec

**Affiliations:** Department of Energy Efficiency, Environmental Monitoring and Alternative Fuel Technologies, Central Mining Institute—National Research Institute, Pl. Gwarków 1, 40-166 Katowice, Poland; n.howaniec@gig.eu

## 1. Introduction

Sustainable management of natural resources remains one of the principal economic and environmental challenges. Considerable efforts are being undertaken worldwide, ranging from the development and implementation of cost-effective and more resource- and energy-efficient production technologies to the recovery, reuse and recycling of waste materials. This is of particular importance in the energy sector, fueling entire economies and having a direct impact on related fundamental challenges of energy security and the green transition [1,2]. The cement production sector is another major economic area and a source of carbon dioxide emissions, focusing the attention of scientists and engineers active in the field and involved in the development of waste-derived cementitious materials [3]. These trends were clearly reflected in the dominant thematic area of this collection.

## 2. Insight Into Recent Advances in Waste Valorization

The compiled works covered, in particular, research devoted to the evaluation of carbon fiber (CF) recovery methods; the application of CF, recovered from retired wind turbine blades, with oil shale ash in cement-based composites, or with steelmaking dust and coal-derived fly ash in the production of geopolymer-based granulates; the use of blast-furnace slag, low-grade calcined clay, limestone and lava in cementitious materials development; the application of H_2_S, potentially derived from biotechnological processing of organic waste, in the synthesis of selenium sulfide; the development of metal oxide nanoparticle-functionalized antibacterial textiles using recycled cotton and polyester yarns; and the preparation and characterization of new layered composite materials based on silicone implant waste, biochar–sulfur composites, and mesoporous silica from bottle-derived soda–lime glass.

Industrial demand for CF components is growing and reflects their increasing use in the production of lightweight and durable materials, in particular for automotive, aerospace and wind turbine applications. This is inherently combined with the need for cost-effective, environmentally friendly and energy-efficient methods for managing CF-derived waste materials [4]. In Contribution 1, by Poranek et al., the results of laboratory tests and a life cycle assessment (LCA) of two solvolysis-based CF composite recovery methods were presented. The first one consisted of ambient pressure solvolysis in 0.5 and 2 L batch reactors connected to a reflux condenser and an inert gas supply tank, using a solution of ethylene glycol and potassium hydroxide [5]. The second one used plasma-enhanced nitric acid solvolysis to increase the CF recovery rate [6]. LCA results showed that the second option, which is still at the development stage, has the highest negative environmental impact compared to CF production from virgin materials, but also the potential for improved performance through optimization. In contrast, the first option offers environmental benefits compared to virgin fiber production.

Another example of the valorization of short carbon fibers (SCFs), widely used in a variety of industrial applications [7], is provided by Kalpokaitė-Dičkuvienė et al. in Contribution 2. SCFs recovered from the pyrolysis of retired wind turbine blades were employed to modify the functional properties of mortar composites, while oil shale combustion fly ash was tested as a partial cement substitute. The effects on hydration phase composition, pore size distribution, specific surface area, compressive and flexural strength, freeze–thaw cycling, and water absorption of composites over a period of up to 120 days were observed, and their environmental performance was assessed. The use of ash resulted in a refined pore structure and an increased volume of reaction products, primarily calcium silicate hydrates (C–S–H), which are critical for cement strength. The mineralogical composition of the modified binder was reported to be the dominant factor in achieving a dense microstructure that enhanced resistance to water ingress and improved mechanical performance under long-term hydration and freeze–thaw exposure. LCA demonstrated that combining complex binders with SCF may mitigate the environmental impact of cement production in terms of global warming potential, terrestrial ecotoxicity, human non-carcinogenic toxicity and fossil resource scarcity. Both waste materials tested were proven suitable for recycling in the development of cementitious materials for selected applications. Considering the potential of the cement and wind turbine markets [8,9], as well as the CO_2_ emissions associated with the power sector and cement industry [3,10], these findings may provide a foundation for the development of next-generation binder systems.

Composites recovered from retired wind turbine blades were also tested as a primary aluminosilicate precursor, while steelmaking dust and coal-derived fly ash were used as additives in the production of geopolymer-based aggregates by Cempa et al. in Contribution 3. The study contributed to current research focused on recycling selected waste materials from the energy sector for the development of construction materials [3,11]. Reactive solids and alkaline activator liquids were mixed and granulated in a single operation using an intensive counter-current mixer. The materials developed were cold-cured at room temperature and characterized after 28 days in terms of grain size distribution, crushing resistance, water absorption, abrasion, and leaching properties. Scanning electron microscopy imaging and energy-dispersive X-ray spectroscopy analysis were also performed. The results indicated that the additives improved the mechanical performance of the aggregates compared to commercial lightweight aggregates and enhanced their microstructure, while effective immobilization of several heavy metals was also achieved.

The next case of waste valorization as a supplementary cementitious material [12] is Contribution 4 by Etcheverry et al., who tested cement blends with ground-granulated blast-furnace slag, low-grade calcined clay, limestone or lava. The reactivity of the blends was tested using isothermal calorimetry. Results showed slightly lower 28-day strength for blends with clay than for those with blast-furnace slag, as well as a strength increase at later ages for blends with lava due to delayed pozzolanic activity. Concrete produced using low-grade calcined clay and lava was reported to achieve compressive strength comparable to that of the reference cement III/A, but exhibited higher capillary porosity, leading to increased water absorption, drying shrinkage, and reduced freeze–thaw resistance.

Contribution 5 by Gong et al. is a comprehensive review on the recycling of another industrial waste, soda residue, whose unique structural properties and cementitious activity make it a promising mineral admixture for cementitious materials, contributing to the development of the green construction sector [13]. The authors concluded that the physicochemical properties of soda residue, its influence on microstructure, and its effect on macro performance enhance the compactness and early strength of cementitious materials through physical filling and chemical activation. However, its chloride content and the associated salt crystallization phenomenon weaken workability and durability. Its successful employment requires precise dosage control, chemical modification, and the use of mineral admixtures. The identified future research needs include the development of composite binder systems, innovations in solid waste-based material preparation, and advancements in desalination technologies to enable the large-scale and environmentally friendly use of the waste concerned.

Solid products derived from the thermochemical processing of apple wood chips under a carbon dioxide atmosphere were tested in the production of biochar–sulfur composites by Syguła et al. in Contribution 6. The effects of the preparation method (using a muffle furnace or electric burner) and sulfur content (in the range of 60–80%) on the mechanical performance of the composites, as potential durable and sustainable materials for emerging applications [14,15], were determined. The results showed that an increase in sulfur content led to an improvement in the ultimate strength of the composites and that the use of an electric burner resulted in composites exhibiting superior strength and reduced brittleness compared to those fabricated in a muffle furnace. The latter effect was attributed to improved sulfur distribution and more effective infiltration of liquid sulfur into the porous biochar structure under electric burner conditions.

The use of bottle-derived soda–lime glass as a sustainable silica source for the synthesis of mesoporous silica, as an alternative to conventional silica precursors, was tested by de Oliveira Lima et al. in Contribution 7. Glass powder was dissolved in sodium hydroxide to form silica gel, which was subsequently applied as a precursor in porous silica synthesis [16]. The structural, morphological, surface, and textural properties of the materials tested were determined. X-ray diffraction analysis confirmed the amorphous nature of all samples, while Fourier-transform infrared spectroscopy spectra indicated successful silica network formation with modifications in bond connectivity. Scanning electron microscopy imaging revealed spherical particles with average diameters of approx. 0.19 ± 0.02 µm for silica gel and 0.15 ± 0.03 µm for mesoporous silica. Zeta potential measurements indicated a negative surface charge and good colloidal stability in aqueous media. On the basis of the specific surface area, it was concluded that the development of porous structure was limited by the solubility of silica gel in acidic media, which prevented ideal condensation on the surface of the surfactant micelles. While the waste glass-derived silica gel was assessed as a promising precursor, the lack of a highly ordered mesostructure indicated the need for more effective control of precursor solubility and pH.

Following the increased interest in developing more sustainable methods of chemical production for the pharmaceutical and cosmetic industries [17,18], Safinazlou et al. presented in Contribution 8, a preliminary feasibility study devoted to the synthesis of selenium sulfide using H_2_S, which can be produced via a biotechnological route from organic waste sources (e.g., fermentation juice from vegetables, spoiled milk, or compost) and naturally occurring mineral SO_4_^2−^ sources (such as mineral water from gypsum-rich soils), as an alternative to the conventional and more complex chemical method [19]. The preliminary results of the study were considered to be promising; however, further testing, optimization, and upscaling are needed before the ecological benefits can be confirmed and economic value realized.

A study contributing to reports on the sustainable management of breast implant waste [20] was reported in Contribution 9 by Kozłowska et al., who examined its potential use as a component of layered composites. Experimental data acquired using attenuated total reflection Fourier-transform infrared spectroscopy, differential scanning calorimetry, and simultaneous thermogravimetric/derivative thermogravimetric analysis coupled with Fourier-transform infrared spectroscopy confirmed that radiation did not affect the structure, thermal properties, or oxidative decomposition behavior of the shell and gel layers of breast silicone implants. The application of a urethane–methacrylate matrix in composite materials affected the crystallization and melting behavior of the waste-derived silicone phase, whose presence, in turn, increased the thermal stability of the composite while decreasing the glass transition temperature, storage modulus, and hardness compared to the matrix alone. The type of implant waste layer used (shell or gel) did not significantly affect the melting and glass transition temperatures, thermal stability, or oxidative decomposition behavior of the developed materials. The potential practical applicability of the layered composites developed and tested within the study was demonstrated.

An experimental work contributing to the research area devoted to the application of recycled materials in functionalized textiles [21] was presented by Faheem et al. in Contribution 10. A preliminary study on the development of metal oxide nanoparticle-functionalized antibacterial textiles using recycled cotton and polyester yarns was performed. Antibacterial finishes were applied at both the yarn and fabric stages to analyze differences in performance. Agar plate qualitative antibacterial analyses proved the presence of antibacterial properties before and after commercial laundering. Thermophysiological comfort properties were reported to be dependent on recycled yarn types and finishing stages. The air permeability of fabric-finished specimens was about 47% lower, while the overall moisture management capability index was 21% higher, than that of yarn-finished specimens. Bursting strength was observed to decrease since the finishing treatments made the yarns stiffer. A more detailed characterization of nanoparticles deposited onto the textile material and the determination of the effects of specific functional nanoparticles on antibacterial performance were mentioned among the potential subjects for future research.

## 3. Summary

This Special Issue provided a platform for communicating recent insights into the implementation of conventional and emerging material-processing techniques and strategies for the recovery and recycling of waste materials of various origin. The majority of the works collected covered the valorization of energy sector-derived waste, largely in the development of cementitious materials. The remaining studies concerned the recycling of a wide range of wastes with the aim of developing functional materials for defined or emerging purposes in the cosmetic, medical, glass or construction industries. The studies presented included material recovery methods, preparation strategies, and assessments of properties determined experimentally using a variety of analytical techniques. The active engagement of researchers representing various scientific disciplines, reflected in the coverage of this Special Issue, proves that the subject remains both topical and promising.
